# Pembrolizumab-induced toxic epidermal necrolysis with limited mucosal involvement

**DOI:** 10.1016/j.jdcr.2025.03.017

**Published:** 2025-04-17

**Authors:** S. Minhaj Rahman, Leah Laageide, Josephine D’Angelo, Anna De Benedetto, Kathleen Mannava, Glynis A. Scott, Alison Moynihan

**Affiliations:** Department of Dermatology, University of Rochester Medical Center, Rochester, New York

**Keywords:** cutaneous toxicity, lung adenocarcinoma, oncodermatology, PD-1 inhibitor, pembrolizumab, toxic epidermal necrolysis

## Introduction

Toxic epidermal necrolysis (TEN) is a rare, life-threatening cutaneous reaction with rapidly progressive, painful bullae and desquamation of at least 30% body surface area of the epidermis and mucosal membranes.^1^ Although TEN is typically induced by sulfonamide and beta-lactam antibiotics, anticonvulsants, allopurinol, and nonsteroidal anti-inflammatory drugs, literature highlights an increasing incidence following immunotherapy targeting programmed death 1 receptor (PD-1) and programmed death ligand 1 (PD-L1), particularly pembrolizumab for lung malignancies, a humanized monoclonal anti-PD-1 IgG4 antibody targeting malignant tumors expressing PD-L1.^1^, ^2^, ^3^, ^4^, ^5^, ^6^ We present a case of pembrolizumab-induced TEN with limited oral erosions, no other mucosal involvement, and rapid and complete re-epithelization following treatment with etanercept and cyclosporine.

## Case report

**Fig 1:**
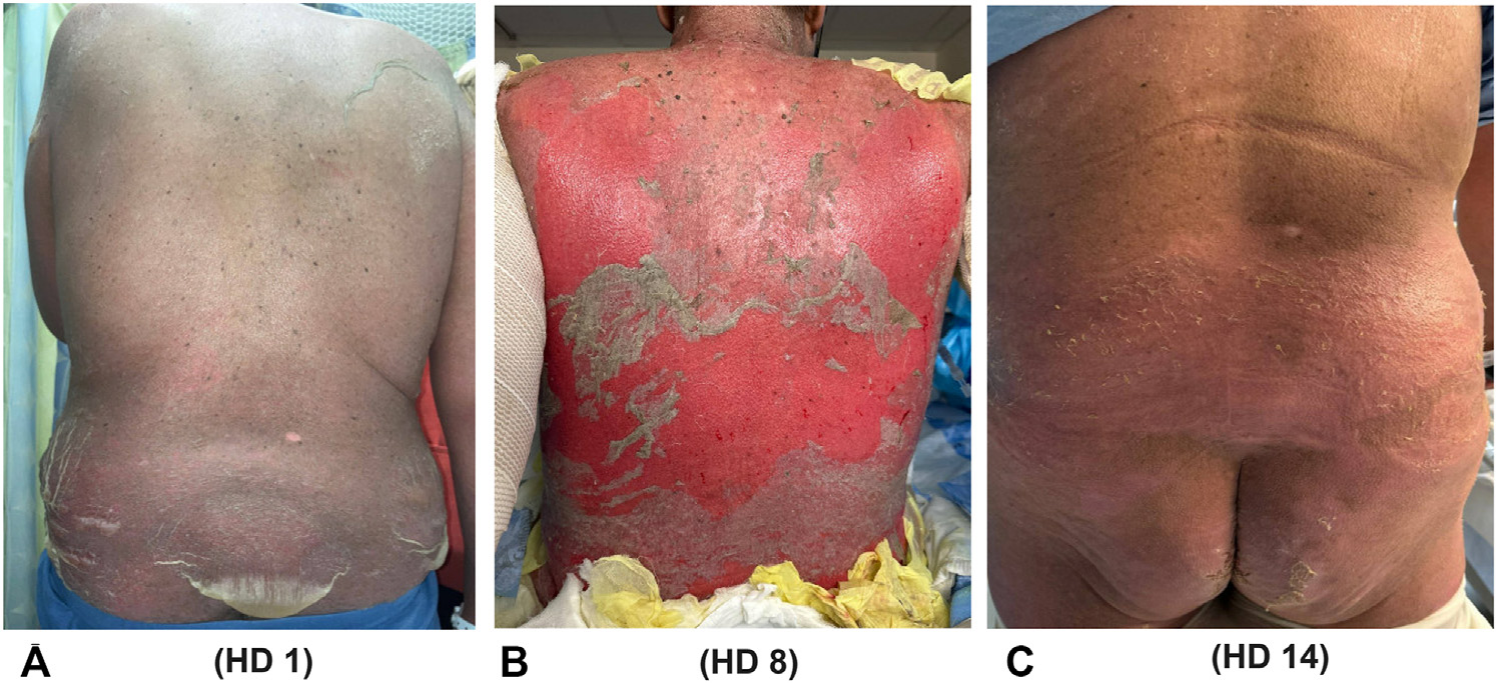
Progression of skin sloughing on the posterior trunk on hospital day (HD) 1 (12/28/24) (**A**), posterior trunk HD 8 (01/05/2024) (**B**), posterior trunk and buttocks on day of discharge, HD 14 (**C**).

A 53-year-old White male with stage IV lung adenocarcinoma was admitted (hospital day (HD) 1) for 24-hours of tachycardia (up to 122 beats-per-minute), diffuse erythema of skin, and tense bullae formation on the lower back that rapidly expanded in approximately 1 day (Fig 1). He initially denied any skin pain, but at the time of presentation he noted increasing discomfort. The patient denied any new additional symptoms. Laboratory findings were notable for a white blood cell count of 15.8 (reference range (RR)): 3.5-11.0 thousand/uL), platelets 697 (RR: 150-450 thousand/uL), blood urea nitrogen (BUN) of 29 (RR: 6-20 mg/dL), lactate 4.3 (RR: 0.5-2.2 mmol/L), alanine transaminase 57 and aspartate aminotransferase 76 (RR: 0-5 U/L), amylase 274 (20-100 U/L), alkaline phosphatase 185 (RR: 40-130 U/L), as well as positive nucleic acid amplification for respiratory syncytial virus. Two 4-mm punch biopsies were obtained from the right lateral thigh for hematoxylin and eosin staining and direct immunofluorescence.

**Fig 2:**
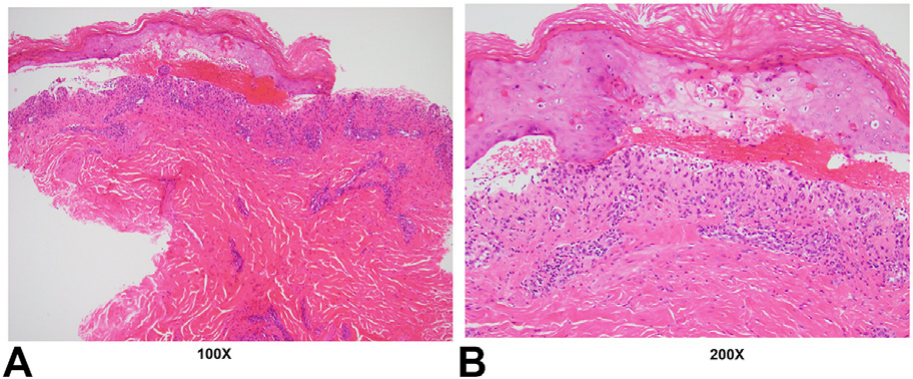
H&E photos the right thigh 4 mm punch biopsy. **A,** - at 100× magnification and (**B**) – at 200× magnification. Biopsy shows vacuolar interface dermatitis leading to subepidermal blister, epidermal necrosis with overlying unremarkable stratum corneum, and a sparse perivascular infiltrate of lymphocytes, histiocytes and few eosinophils. DIF was grossly negative and is not shown in figure. *DIF*, Direct immunofluorescence; *H&E*, hematoxylin and eosin.

On HD2, examination revealed widespread erythema, larger bullae, and peak epidermal detachment of 35% to 40% body surface area. The face and scalp were spared. Pathology was consistent with TEN (Fig 2), with negative DIF. History and examination throughout admission included 2 rapidly healing oral erosions of the inferior tongue but no ocular, intranasal, or genitourinary involvement. Additional medical history included a non-ST elevation myocardial infarction, malignant pleural effusion, bilateral pulmonary, and deep venous thromboembolisms 3-months prior. Social history included 1 standard drink (0.6 oz pure alcohol) per month, but no history of tobacco or illicit drug use.

In the context of his 12D KRAS-mutated and 70% PDL1-positive adenocarcinoma, the patient received a fourth cycle of carboplatin (630 mg), pemetrexed (1100 mg) and pembrolizumab (200 mg) 2 days prior to admission. Medications were suspended in sodium chloride 0.9%/100 mL infusions and cycle 3 was given 7 weeks prior with the addition of zoledronic acid (4 mg/100 ml infusion). He denied any additional new medications and received 3-months of the following therapies prior to the onset of TEN without side effects: oral tablets (apixaban, furosemide, oxycodone, trazodone, pantoprazole, senna, and prochlorperazine), polyethylene glycol, and lidocaine cream.

During hospitalization, patient received IV methylprednisolone 125 mg on HD1, subcutaneous etanercept 50 mg on HD2, and 9 days of oral cyclosporine 4 mg/kg/day (HD 1-10) with rapid re-epithelialization by HD14. Hospital course was complicated by brief fluid overload with minimal oxygen requirements, neutropenia (white blood cell count: 0.8) on HD8 with secondary leukocytosis following 1 dose of filgrastim (300 mcg subcutaneous), and stable malignancy-induced thrombocytosis. Without additional medical sequela, he was discharged to home on HD14. Chemo- and immunotherapy were held following hospitalization; however, outpatient medications were reinitiated without further cutaneous compromise.

## Discussion

We present a case of biopsy-proven TEN in a White male with 5 of 7 SCORTEN risk factors on HD1: age, tachycardia, active malignancy, elevated BUN, and extensive body surface area involvement. Potential triggers include pembrolizumab and pemetrexed. However, the delayed onset of TEN after 4 doses correlates with pembrolizumab’s extended half-life of 14-27 days, supporting its role as the more likely causative agent. Similar cases have shown a median onset of TEN around 11 weeks after initiation of pembrolizumab.^1^ Unlike typical TEN cases with multiple mucous membrane involvement, our patient had minimal, rapidly resolving single mucosal involvement, similar to cases in non-small cell lung cancer patients treated with pembrolizumab. This presentation is distinct from the recently proposed Progressive Immunotherapy-Related Mucocutaneous Eruption (PIRME), which is characterized by a delayed cutaneous reaction to immune checkpoint inhibitors (ICIs), starting as a mild dermatosis and progressing to a generalized bullous eruption with mucosal involvement over days to weeks.^7^ PIRME typically has a slow, progressive onset and a mild, benign course with rapid resolution after systemic therapy, whereas ICI-TEN presents more acutely with rapid progression and severe symptoms, typically without a prodromal rash. Most PIRME cases display mucosal involvement, unlike this patient’s relatively spared mucosa. Both conditions exhibit similar histopathologic features, such as full-thickness epidermal necrosis and subepidermal clefting, but PIRME often shows robust interface dermatitis, which was absent in this patient. Despite ongoing debate about PIRME’s severity, with some cases resulting in mortality,^8^ the acute onset, severe symptoms requiring cyclosporine intervention, limited mucosal involvement, and lack of robust interface dermatitis in this patient suggest a diagnosis of ICI-TEN. Additionally, the features in this case differ from ICI-induced bullous pemphigoid due to rapid onset, severe skin pain without itching, and atypical clinical appearance, despite not obtaining BP 180 of 230 titers.

The unique mucosae-sparing phenotype described here suggests a potential distinct immunopathogenic mechanism driving cutaneous toxicity in ICI-TEN in comparison to conventional drug-induced TEN. Drug-induced TEN implicates multiple distinct pathways, most notably Fas-Fas ligand interactions on keratinocytes, high levels of circulating soluble Fas-Fas ligand, reactive oxygen species-mediated direct keratinocyte toxicity, and, to a lesser extent, direct cell-mediated killing by cytotoxic T cells and natural killer cells. Although the exact pathogenesis of pembrolizumab-induced TEN is unclear, Goldinger et al suggest that PD-1 inhibitors disrupt the interaction of keratinocyte self-antigens and autoreactive CD8+ T-cell activity, which is crucial for T-cell homeostasis in preventing severe cutaneous drug reactions, suggesting that T cells, not keratinocyte-mediated factors or natural killer cells, are the drivers of this reaction.^9^ Variations in the distribution and density of target PD-1 receptor expressions may differ between cutaneous keratinocytes and mucosal epithelial tissues, resulting in differential susceptibilities to immune-mediated reactions. This may explain why ICI-TEN may feature limited mucosal involvement in contrast to the notable mucosal involvement in classical drug-induced TEN.

Aside from discontinuing the offending agent and supportive care, TEN-specific treatment modalities (eg corticosteroids, intravenous immunoglobulin (IVIG), cyclosporine, etanercept, etc.) remain contentious. A recent 2022 Cochrane review established no significant difference in the administration of corticosteroids or IVIG on disease-specific mortality.^10^ Interestingly, our patient attained rapid and complete re-epithelization following treatment with etanercept, a tumor necrosis factor-alpha inhibitor, and cyclosporine, a calcineurin inhibitor. These outcomes align with recent literature on the efficacy of etanercept and cyclosporine in reducing disease-specific mortality compared to corticosteroids and non-cyclosporine treatments, as well as etanercept’s efficacy in reducing hospital stays compared to nonetanercept treatments.^10^, ^11^, ^12^ Cyclosporine is a particularly potent inhibitor of T cell function, which may explain its notable efficacy in this case if ICI-TEN, in which aberrant T cell activity is thought to drive disease. In 6 previously reported cases of ICI-TEN in patients receiving pembrolizumab, clinicians utilized diverse treatments, including high-dose steroids and IVIG in 4 cases, cyclosporine in 3, plasmapheresis in 2, and no administration of etanercept across all cases.^1^, ^2^, ^3^, ^4^, ^5^, ^6^ All but 1 reported patient attained significant or complete re-epithelization within 7-42 days. The lack of uniformity in treatment approach highlights the uncertainty surrounding the ideal management of pembrolizumab-induced TEN. Although there is no consensus regarding treatment of ICI-TEN, our case adds to the limited but growing evidence supporting the use of cyclosporine, showing rapid re-epithelialization without the prolonged need for high-dose corticosteroids or IVIG.

## Conflicts of interest

Dr Benedetto is a consultant for AbbVie, and an investigator for Incytes Crp and Pfizer. Drs Laageide, Angelo, Mannava, Scott, Moynihan, and Author Rahman have no conflicts of interest to declare.
